# Changing trajectories of serum uric acid and risk of non-alcoholic fatty liver disease: a prospective cohort study

**DOI:** 10.1186/s12967-020-02296-x

**Published:** 2020-03-19

**Authors:** Zhimin Ma, Chaonan Xu, Xiaoping Kang, Shan Zhang, Haibin Li, Lixin Tao, Deqiang Zheng, Xiuhua Guo, Xinghua Yang

**Affiliations:** 1 School of Public Health, Capital Medical University, NO. 10 Xitoutiao, Youanmen, Fengtai District, Beijing, 100069 China; 2 Beijing Municipal Key Laboratory of Clinical Epidemiology, NO. 10 Xitoutiao, Youanmen, Fengtai District, Beijing, 100069 China; 3 Medical Engineering Department, Peking University Third Hospital, NO. 49 HuaYuan BeiLu, Haidian District, Beijing, 100191 China; 4 Beijing Xiaotangshan Hospital, NO. 390 Wenquan Street, Xiaotangshan Town, Changping District, Beijing, 102211 China

**Keywords:** Non-alcoholic fatty liver disease, Serum uric acid, Changing trajectory, Dose–response relationship

## Abstract

**Background:**

It is unclear the role of longitudinal trajectory of serum uric acid (SUA) on the development of non-alcoholic fatty liver disease (NAFLD). We aimed to determine whether longitudinal SUA trajectories are associated with the risk of new-onset NAFLD.

**Methods:**

We explored the relationship between SUA trajectories and NAFLD in a cohort including 3822 participants. Individual’s SUA trajectories from 2012 to 2014 were defined using group-based trajectory modeling analysis in four distinct patterns: trajectory 1 (n = 991, 25.93%), trajectory 2 (n = 1421, 37.18%), trajectory 3 (n = 1156, 30.22%), and trajectory 4 (n = 254, 6.67%). The logistic regression model was used to evaluate the association between SUA changing trajectories and subsequent NAFLD until 2016. Dose–response relationship between SUA changing trajectories and NAFLD risk was evaluated through the testing of trajectory groups as a continuous variable.

**Results:**

The 2-year incidence of NAFLD was 13.27%. Compared with trajectory 1, the adjusted odds risk for NAFLD development was in a dose–response relationship as follows: 1.27 (95% CI 0.91–1.78) for trajectory 2, 1.89 (95% CI 1.29–2.75) for trajectory 3, and 2.34 (95% CI 1.43–3.83) for trajectory 4. And this dose–response relationship was not affected by age, sex, and abdominal obesity.

**Conclusions:**

Higher SUA changing trajectory is a risk factor for NAFLD. This finding highlights the importance of paying attention to SUA changing trajectory on the detection and prevention of NAFLD.

## Background

Non-alcoholic fatty liver disease (NAFLD) is a metabolic disorder of the liver and may predispose the affected individual to the development of cirrhosis and hepatocellular carcinoma [1]. Lifestyle changes in developed Western and Asian countries have led to an increase in the incidence of NAFLD. NAFLD becomes the most common cause of chronic liver disease in the world affecting approximately 24% of the global population [2]. And the prevalence of NAFLD in Asian countries is approximately 27.37% [3]. In addition, a meta-analysis based on the Chinese population showed that the prevalence of NAFLD in China is 20.09% [4]. Therefore, NAFLD has become a serious public health problem, and it’s necessary to study the predictive factors.

Besides, a meta-analysis has demonstrated an association between serum uric acid (SUA) and NAFLD, which suggests that increased SUA levels might prompt a physician to screen for NAFLD [5]. Recent studies reported that a high SUA level is a risk factor for NAFLD, and hyperuricemia is a common finding in patients with NAFLD and is independently associated with early histological findings in this clinically relevant condition [6, 7]. However, most of these studies used a single measurement of SUA to predict NAFLD risk, which omitted a possible variability of trajectories of SUA over time. Hence, we conducted a NAFLD cohort study to capture the longitudinal SUA changing trajectory from 2012 to 2014, and to analyse the association between SUA changing trajectory and NAFLD risk until 2016. Besides, we also evaluated the dose–response relationship between SUA changing trajectory and NAFLD risk. This study would suggest the trajectory of SUA over time may provide an important clue to the development of NAFLD.

## Methods

### Study population

**Fig. 1:**
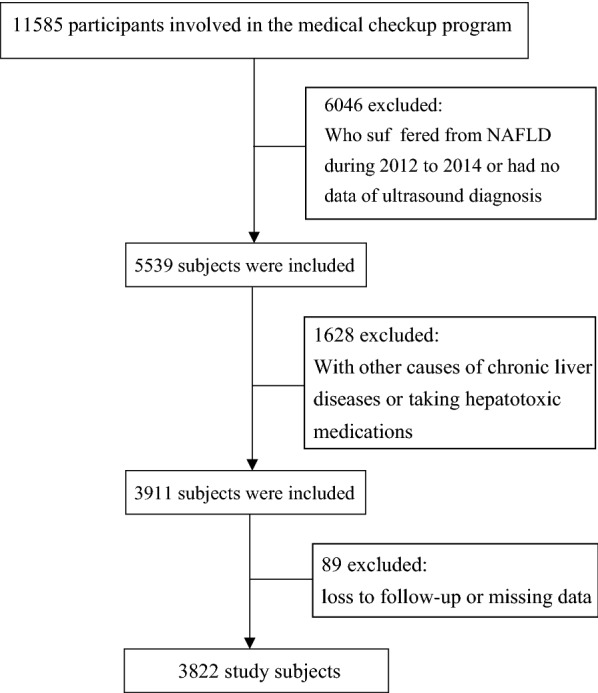
A schematic diagram of the study participants ethics committee approval

This cohort was built using data from the Health Management Cohort (BHMC). The BHMC is a large-scale longitudinal cohort study that investigates the development of metabolic disorders in healthy individuals from urban areas of Beijing [8, 9]. There were 11,585 participants who were recruited to undergo a physical examination in 2012. Because a previous study reported that NAFLD significantly increases the risk of incident hyperuricemia [10], we excluded NAFLD participants during 2012–2014 and analysed the SUA changing trajectories among individuals without NAFLD to avoid the effect of NAFLD on the SUA changing trajectories. We also excluded participants who missed the date of ultrasound diagnosis over 2012–2014, without follow-up data, or had other known causes of chronic liver diseases or taking hepatotoxic medications. Thus, a total of 3822 participants were analyzed in this study (Fig. 1).

### Data collection

The research data were obtained in a unified manner and included a questionnaire, physical examination, laboratory testing, and US assessment. A survey questionnaire was used to collect information on demographic characteristics, lifestyle factors, medication use, and history of diseases. The survey was performed by trained investigators. Dietary habits were categorized as meat-based, vegetable-based, and balanced diet. Drinking status was classified as no, little, moderate, and heavy. Smoking status was considered as those who had smoked ≥ 100 cigarettes during their lifetime. Exercise was categorized into 4 classes: no, low (such as light walking, practicing tai chi, dancing, etc.), moderate (such as jogging, biking, climbing, etc.), and high (such as swimming, playing badminton, jumping rope, etc.). The anthropology examination included testing blood pressure, height, weight, and waist circumference (WC).

The laboratory tests included measurements of SUA, alanine aminotransferase (ALT), aspartate aminotransferase (AST), γ-glutamyl transpeptidase (GGT), total cholesterol (TC), high-density lipoprotein cholesterol (HDL-C), triglyceride (TG), low-density lipoprotein cholesterol (LDL-C), fasting plasma glucose (FPG), blood urea nitrogen (BUN), and serum creatinine (SCr). The venous blood samples were collected after a 12-h overnight fast and obtained before 10:30 a.m. All biochemical assays were conducted in the same laboratory using standard laboratory methods, and were measured an enzymatic method using a chemical analyser (Beckman LX 20, America). The hepatic ultrasound examinations were performed by experienced doctors. Visceral adiposity index (VAI) was calculated to assess visceral adiposity. VAI = (WC (cm)/39.68 + (1.88 × BMI))) × (TC/1.03) × (1.31/HDL-C) for males; and VAI = (WC (cm)/(36.58 + (1.89 × BMI))) × (TC/0.81) × (1.52/HDL-C) for females [11]. Besides, estimated glomerular filtration rate (eGFR) was calculated using the Modification of Diet in Renal Disease (MDRD) equations for Chinese population [12]:

$$\text{eGFR mL}/(\text{min}\cdot{1.73\,{\text{m}^2}})\,=\,186\times [\text{SCr}\,(\upmu{\text{moL}}/{\text{L}})\times {0.011312}^{-1.154}\times{\text{age}}^{-0.203}\times{0.742\,(\text{if}\,\text{female})}\times {1.233\,(\text{if}\,\text{Chinese})}]$$

### Diagnostic criteria

NAFLD was diagnosed based on the Chinese criteria [13]: (1) slight diffuse increase and bright homogeneous echo pattern in the liver parenchyma with normal visualization of the diaphragm, portal, and hepatic vein borders, and a normal hepatorenal echogenicity contrast; (2) diffuse increase in the bright echoes in the liver parenchyma with slightly impaired visualization of the peripheral portal and hepatic vein borders; (3) marked increase in the bright echoes at a shallow depth with deep attenuation, impaired visualization of the diaphragm, and marked vascular blurring; and (4) alcohol consumption was < 140 g/week for men and < 70 g/week for women.

Hyperuricemia was identified as SUA level > 420 μmol/L (7.0 mg/dL) in male and > 360 μmol/L (6.0 mg/dL) in female [14]. The diagnostic criteria for diabetes were based on FPG [15]. An FPG level ≥ 7.0 mmol/L or the use of glucose-lowering drugs was considered as diabetes. According to the harmonized definition of the metabolic syndrome (MS) established in 2009, the MS was identified by requiring the existence of least three or more criteria [16]: (1) abdominal obesity referred to WC ≥ 90 cm in men, WC ≥ 80 cm in women; (2) high TG ≥ 1.7 mmol/L or the use of drug treatment for elevated TG; (3) low HDL-C < 1.0 mmol/L in men, HDL-C < 1.3 mmol/L in women or the use of drug treatment for reduced HDL-C; (4) elevated blood pressure of systolic ≥ 130 mmHg and/or diastolic ≥ 85 mmHg, or the use of an antihypertensive drug treatment; and (5) high FPG ≥ 5.6 mmol/L.

### Statistical analysis

All statistical analysis was performed using SPSS version 22.0 (Chicago, IL) and SAS version 9.4 (SAS Institute, Cary, NC). The two-sided statistical significance level was set at α = 0.05.

Firstly, data were described as the mean ± standard deviation and number with percent frequency for continuous and categorical variables, respectively. Comparisons among groups were conducted by analysis of variance for continuous variables and the Chi square test for categorical variables. Univariate repeated measures ANOVA was preformed to evaluate the changes in SUA over time.

Secondly, semiparametric group-based trajectory modeling (GBTM) was used to characterize the trajectory patterns of SUA from 2012 to 2014 in the study cohort. Briefly, we used the procedure ‘PROC TRAJ macro’ to fit a semiparametric mixture model using the maximum-likelihood method [17]. We empirically compared one, two, three and four group solutions and then optimized the number of subgroups by Bayesian Information Criterion values (close to zero indicating a good fit), where in the shapes of trajectories were determined according to the order of the polynomial (linear, quadratic, cubic, etc.). The optimal number of trajectories and trajectory shapes were determined by the following criteria [18]: (1) improvement in the Bayesian information criterion; (2) no less than 5% membership in each trajectory group; and (3) high group average posterior probabilities (> 0.7).

Thirdly, the association between SUA changing trajectories and the cumulative incidence rate of NAFLD was analyzed using the Cochran-Armitage trend test. We evaluated the relationships between SUA trajectories and NAFLD risk until 2016 using logistic regression analyses. Three models were conducted. Model 1 was a univariate analysis, and model 2 was adjusted for age and sex; as well as model 3 was adjusted for age, sex, drinking, smoking, dietary habits, exercise, BMI, abdominal obesity, VAI, BUN, SCr, eGFR, dyslipidemia, MS, diabetes, hypertension, and use of antidyslipidemia medication. The C statistic was used to assess the discrimination ability of the models. Besides, dose–response relationships for each SUA changing trajectory were evaluated by examining the odds ratio (OR) across trajectory groups, with significance evaluated through the testing of trajectory groups as a continuous variable.

Fourthly, exploratory subgroup and interaction analyses were used to evaluate effect modification in the adjusted models. Participants were stratified according to age, sex, and abdominal obesity because those grouping variables have been reported to be associated with NAFLD risk [19–22]. Age was classified as < 60 and ≥ 60 years. Because a meta-analysis, summarized the prevalence of NAFLD in China, has reported that the prevalence of NAFLD increased with age and decreased after 60 years-of-age among the total population [23].

Finally, three sensitivity analyses were performed to assess the robustness of our results. Elevated ALT, AST, or GGT were considered as surrogate markers of NAFLD. The range of abnormal liver enzymes are as follow: an elevated ALT was a level > 30 U/L for male and > 19 U/L for female, an increased GGT level was a level > 51 U/L for male and > 33 U/L for female [24], as well as an increased AST was a level ≥ 40 U/L [25].

## Results

### The trajectory of SUA

**Fig. 2:**
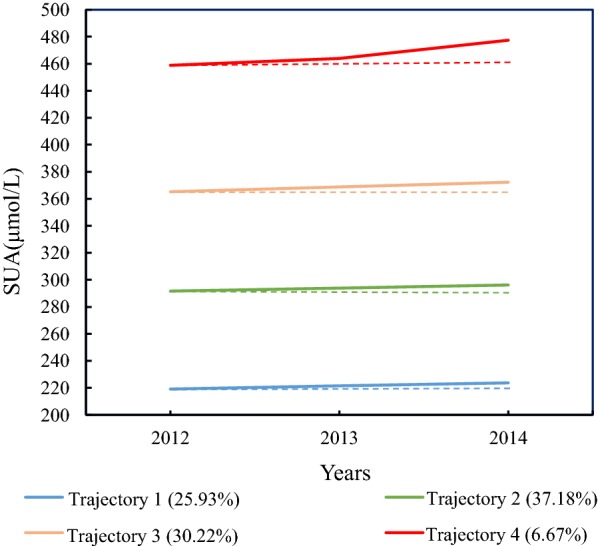
Trajectories of SUA during 2012–2014. The solid lines indicate fitted estimates over 2012-2014, and the dashed lines represent the baseline values. SUA, serum uric acid

The average SUA level from 2012 to 2014 was 307.09 ± 78.4 μmol/L, 306.6 ± 79.9 μmol/L, and 313.33 ± 80.7 μmol/L (Additional file 1: Table 1). And there was a statistically significant change of SUA over time (*F *= 50.277, *P *< 0.01). Furthermore, the quadratic component of the one trajectory by GBTM was significant (Additional file 2: Figure  1), suggesting there was heterogeneity or different change distribution of various persons. Hence, the different combinations of number trajectories (≥ 2) and the order of the polynomial (linear, quadratic, cubic) were analyzed. Finally, four SUA trajectories were identified as the best fitted model by GBTM (Fig. 2 and Additional file 1: Table 1): 25.93% of individuals were classified as having normal baseline with increase (trajectory 1); Baseline SUA was higher in trajectory 2 than that in trajectory 1 but at normal level (37.18%); Trajectory 3 had a higher baseline with moderate increase (30.22%); 6.67% started out with an abnormal baseline (SUA > 420 μmol/L) and then with rapid nonlinear increase (trajectory 4).

### Characteristics of various SUA trajectories at baseline

Table 1 shows the baseline characteristics stratified by SUA trajectories. There was statistical difference in age, sex, drinking, BMI, BUN, SCr, eGFR, VAI among SUA trajectories. Trajectory 4 had the highest frequency of abdominal obesity, dyslipidemia, MS, as well as hypertension.

**Table 1:** Baseline demographic clinical characteristic according to SUA trajectories

Variables	Total (n=3822)	Trajectory 1 (n=991)	Trajectory 2 (n=1421)	Trajectory 3 (n=1156)	Trajectory 4 (n=254)	*P*
Age, years	50.49 ± 15.53	44.45 ± 11.76	51.53 ± 15.04	52.95 ± 16.56	56.95 ± 19.04	< 0.001
Male, n (%)	2109 (55.18)	110 (11.10)	727 (51.16)	1024 (88.58)	248 (97.64)	< 0.001
Dietary habits, n (%)						
Meat-based	430 (11.25)	11 3(11.41)	167 (11.75)	116 (10.03)	34 (13.38)	0.348
Vegetable-based	320 (8.37)	91 (9.18)	124 (8.73)	84 (7.27)	21 (8.27)	
Balanced diet	3072 (80.38)	787 (79.41)	1130 (79.52)	956 (82.70)	199 (78.35)	
Drinking, n (%)						
No	2696 (70.54)	746 (75.28)	979 (68.90)	798 (69.03)	173 (68.11)	0.0171
Little	754 (19.72)	172 (17.36)	288 (20.27)	239 (20.67)	55 (21.65)	
Moderate	301 (7.88)	60 (6.05)	128 (9.01)	90 (7.79)	23 (9.06)	
Heavy	71 (1.86)	13 (1.31)	26 (1.83)	29 (2.51)	3 (1.18)	
Smoking, n (%)	37 (0.97)	14 (1.41)	13 (0.91)	8 (0.69)	2 (0.79)	0.3779
Exercise, n (%)						
No	499 (13.06)	151 (15.24)	178 (12.53)	147 (12.72)	23 (9.06)	0.1826
Low	2388 (62.48)	612 (61.76)	904 (63.61)	709 (61.33)	163 (64.17)	
Moderate	678 (17.74)	170 (17.15)	244 (17.17)	219 (18.94)	45 (17.71)	
High	257 (5.72)	58 (5.85)	95 (6.69)	81 (7.01)	23 (9.06)	
BMI, kg/m^2^	23.19 ± 2.67	22.14 ± 2.66	23.07 ± 2.64	24.01 ± 2.46	24.25 ± 2.26	< 0.001
Abdominal obesity, n (%)	1016 (26.58)	236 (23.81)	377 (26.53)	324 (28.03)	79 (31.30)	< 0.001
Dyslipidemia, n (%)	988 (25.85)	220 (22.20)	341 (24.00)	338 (29.24)	89 (35.04)	< 0.001
MS, n (%)	264 (6.91)	30 (3.03)	78 (5.49)	121 (10.47)	35 (13.78)	< 0.001
Diabetes, n (%)	120 (3.14)	24 (2.42)	54 (3.80)	35 (3.03)	7 (2.76)	0.274
Hypertension, n (%)	863 (22.58)	115 (11.60)	304 (21.39)	349 (30.19)	95 (37.40)	< 0.001
SUA, μmol/L	313.48 ± 80.77	223.27 ± 33.52	296.73 ± 32.67	374.43 ± 38.93	479.72 ± 54.96	< 0.001
ALT, U/L	17.30 ± 11.49	14.40 ± 7.75	17.24 ± 13.69	19.30 ± 10.85	19.80 ± 10.57	< 0.001
AST, U/L	19.20 ± 7.32	17.55 ± 4.87	19.36 ± 9.11	20.15 ± 6.64	20.48 ± 5.75	< 0.001
GGT, U/L	21.23 ± 19.00	15.78 ± 10.19	20.51 ± 17.90	25.16 ± 22.72	28.52 ± 25.31	< 0.001
BUN, mmol/L	5.20 ± 1.43	4.54 ± 1.19	5.18 ± 1.33	5.58 ± 1.36	6.26 ± 1.85	< 0.001
SCr, μmol/L	72.94 ± 16.54	60.59 ± 11.30	70.80 ± 13.44	81.24 ± 13.27	95.12 ± 19.59	< 0.001
eGFR, mL/(min*1.73m^2^)	98.65 ± 21.32	109.46 ± 21.92	99.19 ± 19.55	92.92 ± 18.19	79.78 ± 19.12	< 0.001
VAI	1.37 ± 1.00	1.03 ± 0.75	1.19 ± 1.03	1.44 ± 1.45	1.57 ± 1.25	< 0.001

*SUA* serum uric acid, *BMI* body mass index, *MS* metabolic syndrome, *ALT* alanine aminotransferase, *AST* aspartate aminotransferase, *GGT* γ-glutamyl transpeptidase, *BUN* blood urea nitrogen, *SCr* serum creatinine, *eGFR* estimated glomerular filtration rate, *VAI* visceral fat index

During the 2-year follow-up, 507 participants have developed NAFLD, and the cumulative incidence rate of NAFLD was 13.27%. The incidence of NAFLD from the trajectory 1 to 4 was 6.96%, 10.91%, 19.12%, and 24.41%, respectively. Cochran-Armitage trend test showed that NAFLD incidence was significantly elevated as SUA changing trajectory increased (*z *= 9.976, *P*_trend_ < 0.05).

### Association of SUA changing trajectory and NAFLD Risk

A logistic regression model was conducted to evaluate the relationship between SUA trajectories and NAFLD risk (models 1–3, Table 2). The relationship between SUA trajectories and NAFLD was statistically significant in all models. In the fully adjusted model (model 3) the OR with 95% confidence interval (CI)) for NAFLD in other trajectories compared with the trajectory 1 were 1.27 (95% CI 0.91–1.78, *P *> 0.050), 1.89 (95% CI 1.29–2.75, *P *< 0.050), and 2.34 (95% CI 1.43–3.83, *P *< 0. 01), respectively. Model 3 presented a good discriminatory power with a C statistic of 0.76 (95% CI 0.73–0.77). Besides, the NAFLD risk increases with the SUA changing trajectory increases in those three models (*P*_trend_ < 0.05).

**Table 2:** Association between SUA trajectories and NAFLD risk during follow-up

Models	Trajectory 1	Trajectory 2	Trajectory 3	Trajectory 4	*P*_trend_
Model 1	1.00	1.64 (1.28–2.20)^*^	3.16 (2.38–4.20)^**^	4.32 (2.96–6.29)^**^	< 0.001
Model 2	1.00	1.54 (1.12–2.12)^*^	2.78 (1.96–3.94)^**^	3.79 (2.44–5.88)^**^	< 0.001
Model 3	1.00	1.27 (0.91–1.78)	1.89 (1.29–2.75)^**^	2.34 (1.43–3.83)^**^	< 0.001

Model 1: unadjusted baseline variables. And the C statistic was 0.63 (95% CI 0.6–0.66)

Model 2: adjusted for age and sex. And the C statistic was 0.64 (95% CI 0.61–0.66)

Model 3: adjusted for age, sex, drinking, smoking, dietary habits, exercise, BMI, abdominal obesity, VAI, BUN, SCr, eGFR, dyslipidemia, MS, diabetes, hypertension, and use of antidyslipidemia medication. And the C statistic was 0.76 (95% CI 0.73–0.77)

All data are expressed as OR with 95%CI

*SUA* serum uric acid, *NAFLD* non-alcoholic fatty liver disease, *OR* odds ratio, *CI* confidence interval, *BMI* body mass index, *VAI* visceral fat index, *BUN* blood urea nitrogen, *SCr* serum creatinine, *eGFR* estimated glomerular filtration rate, *MS* metabolic syndrome

^*^P < 0.050, ^**^P < 0.010

### Subgroup and interaction analyses

Table 3 shows that the dose–response association between SUA trajectories and NAFLD risk was evident in aged < 60 years people (*P*_trend_ = 0.0002), but not in aged ≥ 60 years individuals (*P*_trend_ = 0.2167). And this relationship was not significant different in different age group (*P*_interaction_ = 0.4054). Furthermore, the association between NAFLD risk increases with the SUA changing trajectory increases consistent across subgroup analyses by sex (male, female) and abdominal obesity (yes, no).

**Table 3:** Subgroup analyses for the association between SUA trajectories and NAFLD risk

	Trajectory 1	Trajectory 2	Trajectory 3	Trajectory 4	*P*_trend_	*P*_interaction_
Age<60 years	1.00	1.38 (0.95–2.00)	2.03 (1.31–3.15) ^**^	2.70 (1.50–4.86)^**^	0.0002	0.4054
Age≥60 years	1.00	0.83 (0.35–1.94)	1.21 (0.52–2.85)	1.24 (0.45–3.42)	0.2167	
Male	1.00	0.90 (0.46–1.75)	1.22 (0.64–2.33)	1.53 (0.75–3.13)	0.0135	0.2952
Female	1.00	1.31 (0.87–1.96)	2.94 (1.61–5.38) ^**^	5.59 (0.25–7.40)	0.0018	
Non- obesity	1.00	1.25 (0.80–1.95)	1.67 (1.01–2.76) ^*^	1.96 (1.02–3.78)^*^	0.0175	0.6230
Abdominal obesity	1.00	1.33 (0.79–2.24)	2.21 (1.23–3.97) ^**^	2.94 (1.36–6.33)^**^	0.0010	

All analyses were adjusted for age, sex, drinking, smoking, dietary habits, exercise, BMI, abdominal obesity, VAI, BUN, SCr, dyslipidemia, MS, diabetes, hypertension, use of antidyslipidemia medication, and eGFR, except for subgroup variables. Date are shown as OR with 95%CI

*SUA* serum uric acid, *NAFLD* non-alcoholic fatty liver disease, *OR* odds ratio, *CI* confidence interval, *BMI* body mass index, *VAI* visceral fat index, *BUN* blood urea nitrogen, *SCr* serum creatinine, *eGFR* estimated glomerular filtration rate, *MS* metabolic syndrome

^*^P < 0.05, ^**^P < 0.01

### Sensitivity analysis

Several sensitivity analyses were conducted in this study. The first sensitivity analysis assessed the association between SUA changing trajectories and elevated ALT. Adjusted for covariates, the positive association between higher SUA trajectories and subsequent elevated ALT was statistically significant (Additional file 1: Table 3). Second, SUA changing trajectories was not associated with elevated AST (Additional file 1: Table 4). The third analysis indicated that follow-up GGT increases with the SUA changing trajectory increases (Additional file 1: Table 5).

## Discussion

This study assessed the longitudinal SUA changing trajectories from 2012 to 2014 in a Chinese cohort using GBTM analysis. The SUA changing trajectories were classified into four trajectories. Besides, higher SUA changing trajectory was positively associated with NAFLD risk. In addition, subgroup and interaction analyses also suggested the positive association was not moderated by age, sex, and abdominal obesity. Sensitivity analysis also showed that SUA changing trajectory was correlated with suspected of NAFLD based on elevation ALT or GGT, but not AST.

In this study population, the cumulative incidence of NAFLD was 13.27%, which is similar to the 13.5% of the Hong Kong Chinese adult population that develop NAFLD in 3–5 years [26]. And the incidence of NAFLD from the trajectory 1 to 4 was 6.96%, 10.91%, 19.12%, and 24.41%, respectively. Although SUA has long been recognized as an anti-oxidant agent, its chronic elevation has been regarded as detrimental [27]. Our study was in line with this argument. The trend of SUA changes (Fig. 2) indirectly illustrated that people with SUA levels above the abnormal threshold did not take measures to reduce SUA. And the risk for NAFLD was more than two times higher in trajectory 4 than in trajectory 1. Hence, persistent elevation in SUA could be an important clue to the development of NAFLD. The linear dose–response relationship between SUA and NAFLD risk has been demonstrated in two meta-analyses [28, 29]. On the other hand, this study indicated that there was an increasing dose–response relationship between longitudinal SUA changing trajectories and NAFLD risk. Considering these findings, attention should be paid not only to the elevated SUA but also to the higher SUA changing trajectory.

The biochemical role of SUA in NAFLD is poorly understood. The mechanisms might be that elevated SUA results in insulin resistance [30], mitochondrial oxidative stress [27, 31], endoplasmic reticulum stress [32], induced reactive oxygen species [33], and activation of the NLRP3 inflammasome [34]. Those actions increase lipid production and accumulation in the liver, eventually causing the development of NAFLD.

Subgroup analyses revealed the association between SUA changing trajectory and NAFLD incidence was not affected by age and sex. Indeed, the previous studies reported that the relationship between SUA and NAFLD development could be sex-specific [35–38], and that the mechanism behind the sex differences remains unclear. But our study was first to found that SUA changing trajectory was associated with NAFLD incidence independently of sex. Hence, this result and relative mechanism was worthy of further exploration. In addition, subgroup analysis also showed that positive association between SUA changing trajectory and NAFLD development was observed even in non-obesity or abdominal obesity individuals. Obesity is an important risk factor for NAFLD, though, in recent years, it has been reported that NAFLD can also occur in non-obesity individuals, especially in Asian [39, 40].

In the sensitivity analyses, SUA changing trajectory was associated with suspected of NAFLD based on elevation ALT or GGT, but not AST. NAFLD is usually asymptomatic, so diagnosis usually follows the incidental finding of abnormal liver enzymes or steatosis on imaging [41]. Abnormal liver enzymes included raised ALT, AST, and GGT. Compared to other enzymes, ALT is the mostly liver-specific and is more commonly used as a specific marker of hepatocyte damage [25, 42]. Besides, the previous study has reported that SUA is independently associated with elevated ALT, as a surrogate for NAFLD [43]. Our study was in line with this result and external it to the association between SUA changing trajectory with NAFLD development.

A limitation of this study is that NAFLD was diagnosed by ultrasonography, which cannot determine the severity of steatosis in NAFLD. However, ultrasonography is widely used for population-based studies with reasonable accuracy. And sensitivity analyses were performed by using abnormal ALT, AST, and GGT as surrogate markers in this study. But because the sensitivity of ultrasonography is low in diagnosis of mild fatty liver, there may be a proportion of individuals who were in early stage of NAFLD during 2012–2014. And the mild NAFLD may have an impact on the SUA changing trajectories from 2012 to 2014. Therefore, we cannot rule out the possibility of the reverse causality relationship between NAFLD and SUA change. This issue should be answered by further studies enrolling patients diagnosed by liver biopsy. Another limitation is that study participants were only of Asian ethnicity. As genetic background plays an important role in NAFLD and hyperuricemia, it is a minor limitation that we did not include other ethnicities. The third limitation is that we failed to consider the impact of heightened inflammatory state on the association between SUA changing trajectory and NAFLD risk, further study needs to handle the effect of inflammatory factors, such as C-reactive protein.

Despite these limitations, our results provide important insights into the incidence of NAFLD and its relationship with SUA trajectories and have clinical importance for NAFLD prevention in reminding people to pay attention to SUA levels and its changing trajectory. In addition, GBTM does not assume a priori the existence of trajectories of a specific form, while it allows distinctive latent developmental trajectories that can be learned from the data [44, 45]. Hence, this study using GBTM could focus on the changing trajectory of SUA to identify distinct, mutual exclusive group.

## Conclusions

This study demonstrates that SUA changing trajectory was associated with the risk of new-onset NAFLD. Besides, the association was not moderated by age, sex, and abdominal obesity. These findings suggested that people should pour close attention to SUA management and its changing trajectory for the detection and prevention of NAFLD.

## Supplementary information

**Additional file 1: Table S1.** The average SUA during 2012–2014. **Table S2** The shape parameters of SUA changing trajectory. **Table S3** Association between SUA trajectories and follow-up elevated ALT. **Table S4** Association between SUA trajectories and follow-up elevated AST. **Table S5** Association between SUA trajectories and follow-up elevated GGT.

**Additional file 2: Figure S1.** A trajectory of SUA during 2012–2014. SUA, serum uric acid.

## Data Availability

The datasets analysed during the current study are available from Prof. Guo on reasonable request.

## Abbreviations

SUA: Serum uric acid
NAFLD: Non-alcoholic fatty liver disease
OR: Odds ratio
CI: Confidence interval
WC: Waist circumference
ALT: Alanine aminotransferase
AST: Aspartate aminotransferase
GGT: γ-Glutamyl transpeptidase
TC: Total cholesterol
TG: Triglyceride
HDL-C: High-density lipoprotein cholesterol
FPG: Fasting plasma glucose
BUN: Blood urea nitrogen
SCr: Serum creatinine
VAI: Visceral adiposity index
eGFR: Estimated glomerular filtration rate
MS: Metabolic syndrome
BMI: Body mass index
GBTM: Group-based trajectory modeling
