# Supplementary material for: Changing trajectories of serum uric acid and risk of non-alcoholic fatty liver disease: a prospective cohort study
Source: J Transl Med. 2020 Mar 19;18:133. doi: 10.1186/s12967-020-02296-x (PMC7081554; doi:10.1186/s12967-020-02296-x)
Supplement: Supplementary file 1 — Additional file 1: Table S1. The average SUA during 2012–2014. Table S2 The shape parameters of SUA changing trajectory. Table S3 Association between SUA trajectories and follow-up elevated ALT. Table S4 Association between SUA trajectories and follow-up elevated AST. Table S5 Association between SUA trajectories and follow-up elevated GGT. [file 12967_2020_2296_MOESM1_ESM.docx]

**Supplement Table 1 The average SUA during 2012-2014**

|  | **SUA_2012_** | **SUA_2013_** | **SUA_2014_** |
| --- | --- | --- | --- |
|  | 307.09±78.4 | 306.6±79.9 | 313.33±80.7 |
|  |  |  |  |
| **Mauchly’s test** | *x*^2^=1.655 | *P=*0.437 |  |
| **Pillai’s trace** | *F*=50.277 | *P**<*0.01 |  |

Univariate repeated measures ANOVA was conducted to test for the changes in SUA over time.

SUA, serum uric acid;

**Supplement Table 2 The shape parameters of SUA trajectory**

|  | **Trajectory 1** | **Trajectory 2** | **Trajectory 3** | **Trajectory 4** |
| --- | --- | --- | --- | --- |
| **Intercept** | 219.13 | 291.64 | 365.21 | 458.80 |
| **Linear slope** | 2.30 | 2.27 | 3.52 | 0.95 |
| **Quadratic slope** |  |  |  | 4.17 |
| **Group membership probability** | 25.93% | 37.18% | 30.22% | 6.67% |
| **Group average posterior probability** | 0.92 | 0.88 | 0.91 | 0.93 |

SUA, serum uric acid;

**Supplement Table 3 Association between SUA trajectories and follow-up elevated ALT**

| **Models** | **Trajectory 1** | **Trajectory 2** | **Trajectory 3** | **Trajectory 4** | ***P* _trend_** |
| --- | --- | --- | --- | --- | --- |
| Model 1 | 1.00 | 1.02(0.79–1.31) | 0.90(0.69–1.17) | 1.07(0.70–1.62) | 0.1127 |
| Model 2 | 1.00 | 1.47(1.13–1.91) ^**^ | 1.95(1.39–2.73) ^**^ | 2.66(1.64–4.33) ^**^ | <0.001 |
| Model 3 | 1.00 | 1.35(1.03–1.78) ^*^ | 1.71(1.20–2.44) ^**^ | 2.64(1.56–4.50) ^**^ | <0.001 |

Model 1: unadjusted baseline variables. And the C statistic was 0.52(95% CI: 0.49–0.54).

Model 2: adjusted for age and sex. And the C statistic was 0.61(95% CI: 0.58–0.64).

Model 3: adjusted for age, sex, drinking, smoking, dietary habits, exercise, BMI, abdominal obesity, VAI, BUN, SCr, eGFR, dyslipidemia, MS, diabetes, hypertension, and use of antidyslipidemia medication. And the C statistic was 0.67(95% CI: 0.64–0.69).

All data are expressed as OR with 95%CI.

*P < 0.050, **P < 0.010.

SUA, serum uric acid; ALT, alanine aminotransferase; non-alcoholic fatty liver disease; OR, odds ratio; CI, confidence interval; BMI, body mass index; VAI, visceral fat index; BUN, blood urea nitrogen; SCr, serum creatinine; eGFR, estimated glomerular filtration rate; MS, metabolic syndrome.

**Supplement Table 4 Association between SUA trajectories and follow-up elevated AST**

| **Models** | **Trajectory 1** | **Trajectory 2** | **Trajectory 3** | **Trajectory 4** | ***P* _trend_** |
| --- | --- | --- | --- | --- | --- |
| Model 1 | 1.00 | 2.53(0.94–6.84) | 2.77(1.01–7.58) ^*^ | 0.78(0.09–6.70) | 0.2897 |
| Model 2 | 1.00 | 2.66(0.93–7.58) | 2.90(0.89–9.46) | 0.84(0.09–8.08) | 0.4900 |
| Model 3 | 1.00 | 2.59(0.89–7.49) | 2.76(0. 81–9.38) | 0.67(0.06–7.09) | 0.5982 |

Model 1: unadjusted baseline variables; And the C statistic was 0.60(95% CI: 0.52–0.68).

Model 2: adjusted for age and sex; And the C statistic was 0.62(95% CI: 0.53–0.71).

Model 3: adjusted for age, sex, drinking, smoking, dietary habits, exercise, BMI, abdominal obesity, VAI, BUN, SCr, eGFR, dyslipidemia, MS, diabetes, hypertension, and use of antidyslipidemia medication. And the C statistic was 0.69(95% CI: 0.61–0.76).

SUA, serum uric acid; AST, aspartate aminotransferase; non-alcoholic fatty liver disease; OR, odds ratio; CI, confidence interval; BMI, body mass index; VAI, visceral fat index; BUN, blood urea nitrogen; SCr, serum creatinine; eGFR, estimated glomerular filtration rate; MS, metabolic syndrome.

**Supplement Table 5 Association between SUA trajectories and follow-up elevated GGT**

| **Models** | **Trajectory 1** | **Trajectory 2** | **Trajectory 3** | **Trajectory 4** | ***P* _trend_** |
| --- | --- | --- | --- | --- | --- |
| Model 1 | 1.00 | 1.58(1.06–2.35) ^*^ | 1.74(1.16–2.61) ^**^ | 2.20(1.26–3.87) ^**^ | 0.0023 |
| Model 2 | 1.00 | 1.88(1.24–2.85) ^**^ | 2.47(1.51–4.03) ^**^ | 3. 30(1.72–6.33) ^**^ | 0.0001 |
| Model 3 | 1.00 | 1.69(1.10–2.59) ^*^ | 2.09(1.25–3.50) ^**^ | 3.43(1.70–6.92) ^**^ | 0.0007 |

Model 1: unadjusted baseline variables; And the C statistic was 0.56(95% CI: 0.52–0.60).

Model 2: adjusted for age and sex; And the C statistic was 0.58(95% CI: 0.54–0.62).

Model 3: adjusted for age, sex, drinking, smoking, dietary habits, exercise, BMI, abdominal obesity, VAI, BUN, SCr, dyslipidemia, MS, diabetes, hypertension, use of antidyslipidemia medication, and eGFR; And the C statistic was 0.68(95% CI: 0.64–0.71).

SUA, serum uric acid; GGT, γ-glutamyl transpeptidase; non-alcoholic fatty liver disease; OR, odds ratio; CI, confidence interval; BMI, body mass index; VAI, visceral fat index; eGFR, estimated glomerular filtration rate; BUN, blood urea nitrogen; SCr, serum creatinine; MS, metabolic syndrome.
