# Supplementary figures and images for: Changing trajectories of serum uric acid and risk of non-alcoholic fatty liver disease: a prospective cohort study
Source: J Transl Med. 2020 Mar 19;18:133. doi: 10.1186/s12967-020-02296-x (PMC7081554; doi:10.1186/s12967-020-02296-x)

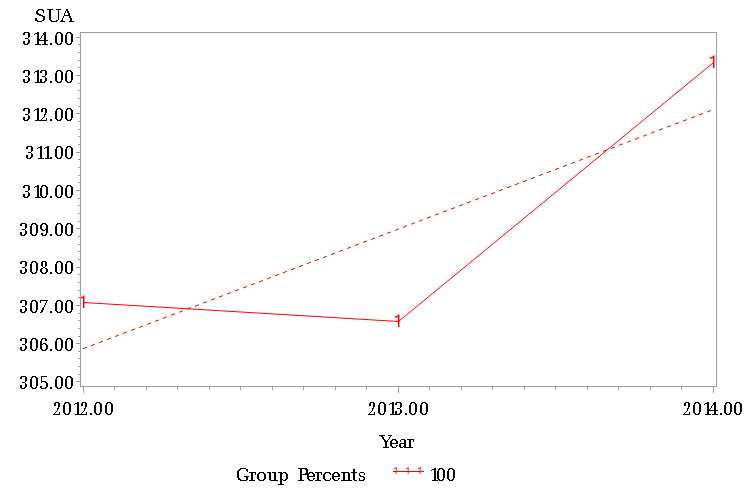

Supplement: Supplementary file 2 — Additional file 2: Figure S1. A trajectory of SUA during 2012–2014. SUA, serum uric acid. [file 12967_2020_2296_MOESM2_ESM.tif]
